# Twin Labor—Double Version

**Published:** 1880-07

**Authors:** P. W. Van Peyma


### TWIN LABOR—DOUBLE VERSION.

BY P. W. VAN PEYMA, M. D.

In the *Buffalo Medical Journal* for September, 1879, is the report of a case of twin labor where both children presented by the arm. This case, occurring in the practice of Dr. Edwin Borck, of St. Louis, was first reported in the *Obstetric Gazette.* As an introduction to the report, we published a table taken from Cazleaux, showing the various presentations obtained in 329 twin labors. In not one of these cases did the first child present transversely. We also remarked at the time that "many other writers fail to mention cases of this character, and otherwise ignore the subject. In regard to the difficulties encountered in turning the first child, it is almost impossible to find anything in treatises upon midwifery."

On the night of May 16th, I was called to see Mrs. S., aged 36, in labor for the sixth time. Her former labors had been easy and wanting in any particular points of interest. A quite intelligent midwife in attendance informed me that the patient had been sick for a number of hours; that the os was dilated to nearly its full size, with the membranes bulging, but that she had been unable to find any part of the child presenting. After having satisfied myself of the truth of these statements, I proceeded to assist the patient. I slowly introduced the hand between the membranes and the walls of the uterus; the umbilical cord, pulsating vigorously, was first encountered; next a hand was felt, and soon, having nearly reached the fundus, both feet were arrived at. After pulling these down a short distance, the hand was passed through the membranes, the child turned, and delivered with little difficulty. It was now discovered that a second child lying transversely and in a separate amniotic sac, still remained. It was easily delivered in a similar manner, the placenta soon following. The children, somewhat undersized, were both alive and apparently healthy.

